# Lessons to be learned: identifying high-risk medication and circumstances in patients at risk for suicidal self-poisoning

**DOI:** 10.1186/s13033-021-00513-8

**Published:** 2022-01-25

**Authors:** Stefanie Geith, Christiane Didden, Christian Rabe, Tobias Zellner, Armin Ott, Florian Eyer

**Affiliations:** 1 Division of Clinical Toxicology and Poison Control Centre Munich, Department of Internal Medicine II, School of Medicine, Technical University of Munich, Munich, Germany; 2 Department of Sociology, University of Munich (LMU), 80801 Munich, Germany; 3 Staburo GmbH, Aschauer Straße 26b, 81549 Munich, Germany

**Keywords:** Suicide, Self-poisoning, Epidemiology, Prevention, High-risk medication

## Abstract

**Background:**

Although the total number of suicides decreased since the beginning of the 1980s, the number of suicide-related behaviors using self-intoxication increased. Therefore, research on the characteristics of individuals committing self-intoxication becomes of growing importance for risk assessments and the development of preventive measures.

**Methods:**

In this prospective, observational, monocentric cohort study, all incoming calls at our Poisons Control Centre reporting suicide-related behaviors through self-intoxication, were analyzed via a standardized questionnaire over 12 months. Both univariate and bivariate analyses were performed.

**Results:**

1238 cases of deliberate intoxication were included in the study. The majority of cases occurred in the age group between 18 and 44 (n = 607/49%), two-thirds were female (n = 817/66%). The main substances used were antidepressants (n = 420/34%), peripheral analgesics (n = 322/26%) and neuroleptics (n = 282/23%). The majority of patients ingested substances from their prescribed medication (n = 640/82%) with the highest proportion in those aged over 64 years (n = 72/113; 91%, p < 0.001). Substance use was reported for the minority of patients (n = 175/23%). For 704 cases (79%), a psychiatric disorder was documented. Factors associated with recurrent suicide-related behaviors were an underlying psychiatric disorder (OR = 6.2; 95% CI 3.8–10.4), substance use (OR = 2.4; 95% CI 1.5–3.8), and ingestion of neuroleptics (OR = 2.1, 95% CI 1.4–3.0) or antidepressants (OR = 1.6; 95% CI 1.2–2.3).

**Conclusion:**

This study might contribute to identifying individuals with an increased risk of suicide-related behaviors by deliberate intoxication and to developing preventive strategies for future suicide attempt(s).

**Supplementary Information:**

The online version contains supplementary material available at 10.1186/s13033-021-00513-8.

## Background

The total number of suicides decreased in Germany since the beginning of the 1980s [1]. Still, suicide is a more frequent cause of death than accidents, illicit drug abuse, acquired immunodeficiency syndrome, and murder combined [2]. After hanging, self-intoxication is the second most common method for suicide with increasing numbers of cases [3].

This emphasizes the importance of collecting reliable epidemiological data about characteristics of self-intoxication in the context of suicide-related behaviors (SRB) as defined by Silverman et al. [4]. We agreed on this nomenclature as there are no universally accepted or operationalized definitions regarding suicidality, and irrespective of this, the intention to die in particular could often not be clearly elicited. This definition seemed particularly appropriate for our study as it also subsumes, besides suicide attempt and suicide, self-harm and suicidal behavior with undetermined intention to die under the generic term SRB. Identification of characteristics of individuals displaying SRB could allow developing better preventive strategies, thereby avoiding preventable deaths. Current information on the characteristics of individuals showing SRB through self-intoxication is limited. Available studies focus either on information on substances used [5], substances used according to age group [6] or on individuals explicitly admitted to emergency care [7–9]. In another study the underlying psychiatric disorders are focused on [10]. Further studies investigate more closely on the characteristics of individuals committing repeated suicide attempts [11]. Accordingly, studies at hand focus on specific types of patients or specific parameters while our study presents information on unselected patients, thus offering information applicable to a broader range of subjects. The only study including parameters that are similar to the ones collected in the study at hand presents information collected between 1985 and 1997 [12].

In this study, the incoming calls at the Munich Poison Control Centre (PCC) were screened to prospectively collect data from a non-selected population in Germany. The Munich PCC established in 1963 by the Free State of Bavaria, is one of seven PCCs in Germany and part of the Department of Clinical Toxicology of a tertiary university hospital in Bavaria. It is primarily responsible for providing advice on poisoning in Bavaria, covering a population of around 13.2 million (corresponding to 16% of the population of Germany). With around 43,000 enquiries per year the PCC is one of Germany’s larger institutions and is contacted by lay people (50%) as well as by clinicians (30%) and general practitioners (15%) seeking advice on poisonings of any kind. These data present a unique profile of SRB via self-intoxication within the German population, offering real-world evidence on characteristics of self-intoxicated suicides and suicide attempts.

## Methods

### Design and setting

**Fig. 1:**
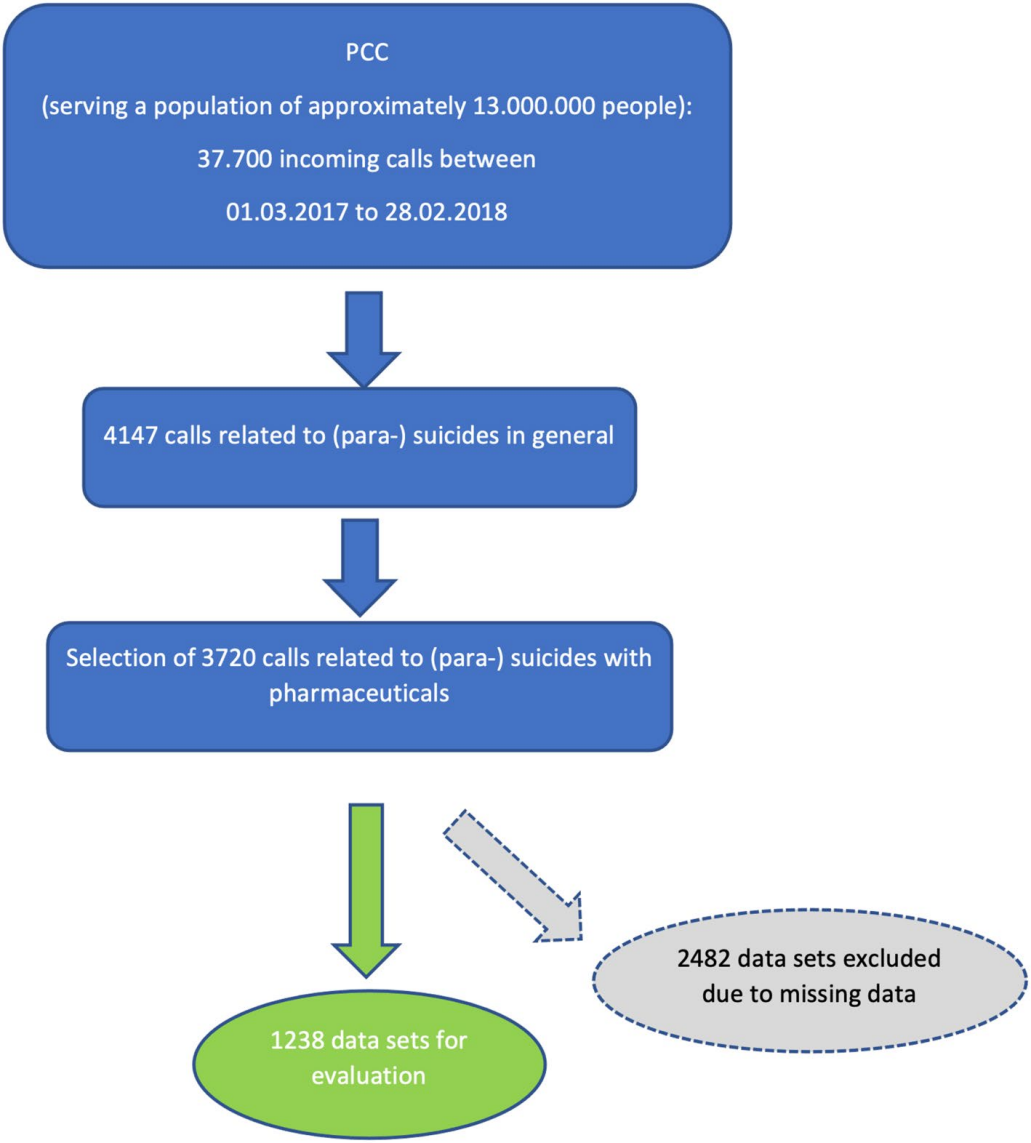
Data flow of incoming calls to Poisons Control Centre (PCC)

The study was set up as a prospective, observational monocentric cohort study. All incoming calls to our PCC (1st March 2017 to 28th February 2018) reporting SRB through self-intoxication, independent of the outcome (fatal/non-fatal), were included (Fig. 1). These calls were initiated by emergency physicians, emergency medical services personnel, emergency and critical care physicians, and occasionally by laypersons who were entrusted with the emergency care of persons with (para-) suicidal intoxications. The data were collected by trained poison specialists, thus ensuring data quality. The study protocol was approved by the institutional review board (IRB) of the University Hospital (589/16 S). All methods were carried out in accordance with relevant guidelines and regulations.

### Data collection and inclusion criteria

The calls were documented on a standardized protocol including type and amount of the ingested substance, time of ingestion, age, and gender of the patient as well as symptoms. Non-opioid analgesics, including ibuprofen, naproxen, diclofenac, acetylsalicylic acid, paracetamol, and metamizole were subsumed under the group “peripheral analgesics”. Furthermore, the existence of an accompanying psychiatric disorder (PD) and previous SRB was enquired. If a PD was present, it was assigned to the diagnostic categories of the 10th revision of the International Statistical Classification of Diseases and Related Health Problems (ICD-10). For the purpose of this study, questions about accessing the toxins, co-ingestion of ethanol or drugs, substance use disorder, history of suicide attempts and alleged reasons for the suicide attempt were added to the PCC’s standard protocol (see Additional file 1). At least two of these items as well as two items of the baseline characteristics (age, gender and ingested substance) were required for inclusion of the case. Due to the collection of pseudonymized data only, the requirement for patient informed consent was waived. Since the data were collected in a real world setting and were often only incomplete due to the treatment urgency, many cases were excluded in advance because of the fragmentary information available.

Nonetheless, the callers were informed about the study and its content before being asked about the parameters of interest.

### Statistics

Categorical variables are presented by absolute and relative frequencies referring to evaluable cases in each analysis. Group comparisons were performed by χ^2^-tests. If the number of expected cell counts was smaller than five, Fisher’s exact test was used.

The level of statistical significance was assumed to be p ≤ 0.05. Because of the exploratory nature of this study, we did not adjust for multiple testing. Statistical analysis was performed using IBM SPSS Statistics for Windows, version 25 (IBM Corp., Armonk, N.Y., USA) and R version 3.5.2 (R Foundation for Statistical Computing, Vienna, Austria).

Multiple answers were possible regarding the ingested substances as well as the sources of supply. In this study, we generated binary variables (yes/no) for each substance as well as for each source of supply and ICD-10 classification for PD. Ranging from 12 to 95 years, the observed variation in age is considerable. Therefore, four age groups have been defined for evaluation purposes (< 18 years, 18–44 years, 45–64 years and > 64 years).

## Results

3720 cases showing SRB were recorded during the survey period. Each case represents one instance of SRB. 2482 cases were excluded due to incomplete data so that 1238 cases were included in the analysis (see Fig. 1). For the different sub-analyzes, the exact number of cases may vary due to missing data. For each analysis presented below, 100% refers to the totality of cases for which the respective parameter were available.

### Gender-specific outcomes

The majority of cases were female (n = 817; 66%). The proportions of the age groups differed across gender (p < 0.001). The majority of cases in both gender groups were between 18 and 44 years old. However, almost 13% (n = 98) of the female population were < 18 years old, whereas in the male population only 5% (n = 18) were < 18 years old.

**Fig. 2:**
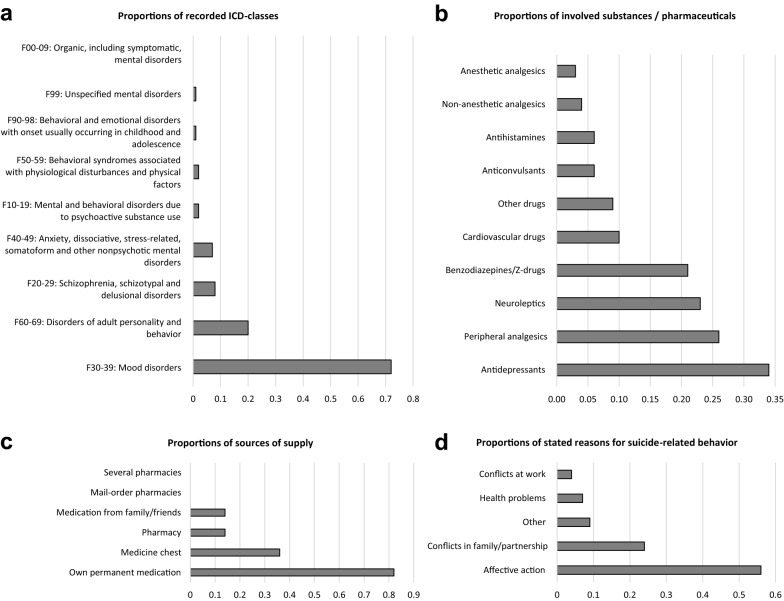
Overview of psychiatric disorders, frequently used substances, sources of supply, and reasons for suicide. **a** psychiatric disorders diagnosed according to the ICD-10 classification, **b** most frequently used substances, **c** sources of supply and **d** most frequent reasons for SRB. Multiple answers were possible

704 cases (79%) had a diagnosed PD, and mood disorders were the dominant PD (Fig. 2a). However, a gender-specific difference with regard to the frequency of a documented PD (n = 491; 81% of all female cases vs. n = 211; 74% of all male cases, p = 0.03) was detected.

13 patients (1%) had a diagnosed substance use disorder according to ICD-10. However, substance use was documented in 174 patients (23%, including 13 patients with a documented substance use disorder). Substance use history was significantly more often documented in men (n = 84, 33%) than in women (n = 90, 18%, p =  < 0.001). Alcohol was the most frequently used addictive substance in both groups (n = 115; 15.2%; see Table 1).

**Table 1:** Clinical characteristics of suicide attempters with respect to gender distribution

	Gender				
	Male	Female	Total	p-Value	Missing data
	n (%)	n (%)	n (%)		
Age groups					
< 18	18 (4.6)	98 (12.9)	116 (10.1)		
18–44	216 (55.2)	391 (51.3)	607 (52.6)		
45–64	112 (28.6)	206 (27.0)	318 (27.6)		
> 64	45 (11.6)	67 (8.8)	112 (9.7)		
Total	391 (100.0)	762 (100.0)	1153 (100.0)	< 0.001	85
Psychiatric disorder					
Yes	211 (74.3)	491 (80.9)	702 (78.8)		
No	73 (25.7)	116 (19.1)	189 (21.2)		
Total	284 (100.0)	607 (100.0)	891 (100.0)	0.031	347
Substance use disorder					
Alcohol	54 (21.2)	61 (12.1)	115 (15.2)		
Illicit drugs	20 (7.8)	13 (2.6)	33 (4.4)		
Multiple addictive substances	2 (0.8)	3 (0.6)	5 (0.7)		
Pharmaceuticals	8 (3.1)	13 (2.6)	21 (2.8)		
None	171 (67.1)	413 (82.1)	584 (77.0)		
Total	255 (100.0)	503 (100.0)	758 (100.0)	< 0.001	480
History of SRB					
Yes	98 (43.9)	226 (45.8)	324 (45.2)		
No	126 (56.3)	267 (54.2)	393 (54.8)		
Total	224 (100.0)	493 (100.0)	717 (100.0)	0.659	521
Reasons for SRB					
Affective action	52 (51.0)	142 (57.0)	194 (55.3)		
Health problems	11 (10.8)	15 (6.0)	26 (7.4)		
Conflicts at work	7 (6.9)	6 (2.4)	13 (3.7)		
Conflicts with family/partner	23 (22.5)	63 (25.3)	86 (24.5)		
Others	9 (8.8)	23 (92.)	32 (9.1)		
Total	102 (100.0)	249 (100.0)	351 (100.0)	0.156	887
Substances					15
Peripheral analgesics					
Yes	120 (29.0)	201 (24.8)	321 (26.2)		
No	294 (71.0)	608 (75.2)	902 (73.8)		
Total	414 (100.0)	809 (100.0)	1223 (100.0)	0.137	
Antidepressants					
Yes	131 (31.6)	289 (35.7)	420 (34.3)		
No	283 (68.4)	520 (64.3)	803 (65.7)		
Total	414 (100.0)	809 (100.0)	1223 (100.0)	0.174	
Neuroleptics					
Yes	80 (19.3)	202 (25.0)	282 (23.1)		
No	334 (80.7)	607 (75.0)	941 (76.9)		
Total	414 (100.0)	809 (100.0)	1223 (100.0)	0.032	
Benzodiazepines, Z-drug					
Yes	85 (20.5)	163 (20.1)	248 (20.3)		
No	329 (79.5)	646 (79.9)	975 (79.7)		
Total	414 (100.0)	809 (100.0)	1223 (100.0)	0.934	
Others					
Yes	148 (35.7)	256 (31.6)	404 (33.0)		
No	266 (64.3)	553 (68.4)	819 (67.0)		
Total	414 (100.0)	809 (100.0)	1223 (100.0)	0.149	
Co-ingestion					
Alcohol	137 (40.9)	186 (28.6)	323 (32.8)		
Illicit drugs	6 (1.8)	4 (0.6)	10 (1.0)		
None	192 (57.3)	460 (70.8)	652 (66.2)		
Total	335 (100.0)	650 (100.0)	985 (100.0)	< 0.001	253
Own medication					
Yes	218 (85.2)	419 (80.0)	637 (81.7)		
No	38 (14.8)	105 (20.0)	143 (18.3)		
Total	256 (100.0)	524 (100.0)	780 (100.0)	0.097	458

### Age group-specific outcomes

The age group-specific differences of the three most commonly ingested substance groups (peripheral analgesics, neuroleptics, benzodiazepines/Z-drugs) (Fig. 2b) were statistically significant (see Table 3). For the age group < 18 years, the usage of peripheral analgesics was highest (n = 40; 35%), while the > 64-year-olds used benzodiazepines/Z-drugs most frequently (n = 37; 33%). The most common source of supply in all age groups was the patient’s own medications (Fig. 2c), yet the older the age the higher the proportion (p < 0.001). A PD was documented less frequently in the > 64-year-old group (n = 48; 59%, p < 0.001).

Furthermore, the distribution of suspected substance use disorder varied with age (p < 0.001). The 45 to 64-year-olds showed the highest prevalence with around one third (n = 65; 33%). Except for those < 18 years, the most frequently documented addictive substance in total was ethanol.

Age group-specific differences (p < 0.001) were found regarding the reasons for SRB. Except for the oldest group, where health problems were reported most frequently, an affective action was dominant in all other age groups (Fig. 2d, see also Table 2 and for the pairwise comparisons Additional file 2).

**Table 2:** Clinical characteristics of suicide attempters with respect to age group distribution

	Age Groups						
	< 18	18–44	45–64	> 64	Total	p-Value	Missing data
	n (%)	n (%)	n (%)	n (%)	n (%)		
Psychiatric disorder							
Yes	71 (78.9)	367 (80.7)	189 (81.8)	48 (59.3)	675 (78.8)		
No	19 (21.1)	88 (19.3)	42 (18.2)	33 (40.7)	182 (21.2)		
Total	90 (100.0)	455 (100.0)	231 (100.0)	81 (100.0)	857 (100.0)	< 0.001	381
Substance use disorder							
Alcohol	1(1.1)	53 (14.4)	52 (26.7)	8 (11.3)	114 (15.8)		
Illicit drugs	2 (2.3)	25 (6.8)	4 (2.1)	0 (0.0)	31 (4.3)		
Multiple addictive substances	0 (0.0)	4 (1.1)	1 (0.5)	0 (0.0)	5 (0.7)		
Pharmaceuticals	0 (0.0)	11 (3.0)	8 (4.1)	2 (2.8)	21 (2.9)		
None	85 (96.6)	276 (74.8)	130 (66.7)	61 (85.9)	552 (76.3)		
Total	88 (100.0)	369 (100.0)	195 (100.0)	71 (100.0)	723 (100.0)	< 0.001	515
History of SRB							
Yes	34 (40.0)	182 (51.3)	76 (45.0)	23 (31.9)	315 (46.3)		
No	51 (60.0)	173 (48.7)	93 (55.0)	49 (68.1)	366 (53.7)		
Total	85 (100.0)	355 (100.0)	169 (100.0)	72 (100.0)	681 (100.0)	0.012	557
Reasons for SRB							
Affective action	23 (57.5)	97 (56.1)	57 (63.3)	9 (27.3)	186 (55.4)		
Health problems	0 (0.0)	9 (5.2)	5 (5.6)	11 (33.3)	25 (7.4)		
Conflict at work	5 (12.5)	7 (4.0)	1 (1.1)	0 (0.0)	13 (3.9)		
Conflict with family/partner	11 (27.5)	45 (26.0)	20 (22.2)	5 (15.2)	81 (24.1)		
Others	1 (2.5)	15 (8.7)	7 (7.8)	8 (24.2)	31 (9.2)		
Total	40 (100.0)	173(100.0)	90 (100.0)	33 (100.0)	336 (100.0)	< 0.001	902
Substances							86
Peripheral analgesics							
Yes	40 (34.5)	175 (28.8)	67 (21.1)	26 (23.2)	308 (26.7)		
No	76 (65.5)	432 (71.2)	250 (78.9)	86 (76.8)	844 (73.3)		
Total	116 (100.0)	607 (100.0)	317 (100.0)	112 (100.0)	1152 (100.0)	0.013	
Antidepressants							
Yes	37 (31.9)	211 (34.8)	118 (37.2)	32 (28.6)	398 (34.5)		
No	79 (68.1)	396 (65.2)	199 (62.8)	80 (71.4)	754 (65.5)		
Total	116 (100.0)	607 (100.0)	317 (100.0)	112 (100.0)	1152 (100.0)	0.37	
Neuroleptics							
Yes	29 (25.0)	154 (25.4)	72 (22.7)	15 (13.4)	270 (23.4)		
No	87 (75.0)	453 (74.6)	245 (77.3)	97 (86.6)	882 (76.6)		
Total	116 (100.0)	607 (100.0)	317 (100.0)	112 (100.0)	1152 (100.0)	0.05	
Benzodiazepines/Z-drugs							
Yes	5 (4.3)	114 (18.8)	76 (24.0)	37 (33.0)	232 (20.1)		
No	111 (95.7)	493 (81.2)	241 (76.0)	75 (67.0)	920 (79.9)		
Total	116 (100.0)	607 (100.0)	317 (100.0)	112 (100.0)	1152 (100.0)	< 0.001	
Others							
Yes	39 (33.6)	187 (30.8)	108 (34.1)	47 (42.0)	381 (33.1)		
No	77 (66.4)	420 (69.2)	209 (65.9)	65 (58.0)	771 (66.9)		
Total	116 (100.0)	607 (100.0)	317 (100.0)	112 (100.0)	1152 (100.0)	0.135	
Co-ingestion							
Alcohol	5 (5.2)	186 (37.8)	101 (40.6)	14 (15.1)	306 (32.9)		
Illicit drugs	1 (1.0)	8 (1.6)	1 (0.4)	0 (0.0)	10 (1.1)		
None	90 (93.8)	298 (60.6)	147 (59.0)	79 (84.9)	614 (66.0)		
Total	96 (100.0)	492 (100.0)	249 (100.0)	93 (100.0)	930 (100.0)	< 0.001	308
Own medication							
Yes	50 (60.2)	297 (80.7)	181 (87.0)	72 (91.1)	600 (81.3)		
No	33 (39.8)	71 (19.3)	27 (13.0)	7 (8.9)	138 (18.7)		
Total	83 (100.0)	368 (100.0)	208 (100.0)	79 (100.0)	738 (100.0)	< 0.001	500

### Repeated instances of SRB

In 325 out of 718 (45%) cases with complete data, at least one prior instance of SRB was recorded. The frequency of repeated instances of SRB varied with age (see Table 2﻿). Moreover, observations with prior instances of SRB suffered more often from a PD (p < 0.001) and from a suspected substance use disorder than those without prior instances of SRB (p < 0.001). They also showed an increased use of neuroleptics (p < 0.001) and antidepressants (p = 0.003) and a less frequent use of peripheral analgesics (p = 0.034) (see Table 3).

**Table 3:** History of SRB via self-intoxication. Assessment of different implications on repeated SRB

	History of SRB				
	Yes n (%)	No n (%)	Total n (%)	p-Value	Missing data
Psychiatric disorder					
Yes	257 (91.1)	201 (62.2)	458 (75.7)		
No	25 (8.9)	122 (37.8)	147 (24.3)		
Total	282 (100.0)	323 (100.0)	605(100.0)	< 0.001	633
Substance use disorder					
Alcohol	45 (18.3)	23 (7.5)	68 (12.3)		
Illicit drugs	11 (4.5)	9 (2.9)	20 (3.6)		
Multiple addictive substances	1 (0.4)	2 (0.6)	3 (0.5)		
Pharmaceuticals	8 (3.3)	5 (1.6)	13 (2.3)		
None	181 (73.6)	269 (87.3)	450 (81.2)		
Total	246 (100.0)	308 (100.0)	554 (100.0)	< 0.001	684
Reasons for SRB					
Affective action	56 (58.9)	87 (55.1)	143 (56.5)		
Health problems	8 (8.4)	12 (7.6)	20 (7.9)		
Conflict at work	4 (4.2)	7 (4.4)	11 (4.3)		
Conflict with family/partner	17 (17.9)	43 (27.2)	60 (23.7)		
Others	10 (10.5)	9 (5.7)	19 (7.5)		
Total	95 (100.0)	158 (100.0)	253 (100.0)	0.366	985
Substances					523
Peripheral analgesics					
Yes	77 (23.8)	122 (31.2)	199 (27.8)		
No	247 (76.2)	269 (68.8)	516 (72.2)		
Total	324 (100.0)	391 (100.0)	715 (100.0)	0.034	
Antidepressants					
Yes	120 (37.0)	103 (26.3)	223 (31.2)		
No	204 (63.0)	288 (73.7)	492 (68.8)		
Total	324 (100.0)	391 (100.0)	715 (100.0)	0.003	
Neuroleptics					
Yes	91 (28.1)	62 (15.9)	153 (21.4)		
No	233 (71.9)	329 (84.1)	562 (78.6)		
Total	324 (100.0)	391 (100.0)	715 (100.0)	< 0.001	
Benzodiazepines/Z-drugs					
Yes	66 (20.4)	82 (21.0)	148 (20.7)		
No	258 (79.6)	309 (79.0)	567 (79.3)		
Total	324 (100.0)	391 (100.0)	715 (100.0)	0.916	
Others					
Yes	121 (37.3)	119 (30.4)	240 (33.6)		
No	203 (62.7)	272 (69.6)	475 (66.4)		
Total	324 (100.0)	391 (100.0)	715 (100.0)	0.051	
Co-ingestion					
Alcohol	93 (33.3)	94 (29.1)	187 (31.1)		
Illicit drugs	2 (0.7)	3 (0.9)	5 (0.8)		
None	184 (65.9)	226 (70.0)	410 (68.1)		
Total	279 (100.0)	323 (100.0)	602 (100.0)	0.544	636
Own medication					
Yes	185 (86.4)	199 (73.7)	384 (79.3)		
No	29 (13.6)	71 (26.3)	100 (20.7)		
Total	214 (100.0)	270 (100.0)	484 (100.0)	0.001	754

## Discussion

This study has six key findings. First, the majority of instances of SRB occurred in the age-group between 18 and 44. Second, approximately two-thirds of the reported cases were women. Third, antidepressants, peripheral analgesics and neuroleptics were the main substances used for self-intoxication. Fourth, most often, the substances came from the patient's own medication. Fifth, although the use of addictive substances played a relevant role, the majority of cases had no suspected substance use disorder. The most commonly abused substance was ethanol. Sixth, factors associated with recurrent instances of SRB were: (i) an underlying PD; (ii) suspected substance use disorder; (iii) the ingestion of antidepressants or neuroleptics.

Two thirds of the total analyzed cases were female, and the proportion of females was particularly high among the < 18-year-olds, which is in line with data from other studies [6, 12–16]. Spiller et al. showed that females constituted nearly 71% of all cases of self-intoxication in patients younger than 25 years [16]. The high proportion of females may surprise, as the majority of completed suicides is performed by males [17–19]. However, both our study and the study conducted by Spiller et al. focus on self-intoxication, which is the preferred method for females when displaying SRB [17, 20]. Furthermore, the present study shows that most cases of self-intoxication are performed in the age group between 18 and 44 years, which is in accordance with further studies on this subject [21].

Antidepressants constitute the most commonly used substance group followed by peripheral analgesics. The latter were predominantly used by younger individuals, which is also reflected in the existing literature [6, 22, 23]. In contrast to many other studies, in which benzodiazepines are usually ranked among the three most popular substances [7, 13, 21, 24, 25], they ranged only on fourth place in our investigation. A similar pattern could be detected regarding paracetamol, which ranged as the most common single substance in other studies ([7, 25], and also Additional file 3) but was only the fourth common in our study. Instead, ibuprofen was the most common single substance, which is important as ibuprofen has a high mortality rate (in large overdoses) despite its perceived harmlessness [26, 27]. As the study at hand presents a profile of the characteristics of individuals displaying SRB through self-intoxication in Germany, the relatively uncommon use of benzodiazepines and paracetamol might reflect a more geographic preference or could indicate a change in prescription behavior.

Almost 80% of the cases had a prevalence of PD, which is higher compared to data found in literature [13, 20, 28]. This might be partly biased by the method of data acquisition, as the diagnosis of a PD was mostly not recorded by a psychiatrist.

Females seemed to suffer more often from an accompanying PD. This finding is in line with data from Ghazinour et al. [13]. In contrast, Prescott et al. found no gender-specific difference and Mauri et al. found gender-specific differences concerning specific PDs only [20, 28].

Focusing more closely on the types of PD, mood disorders dominated by far. This corresponds to findings from other studies showing that 65–78% of SRBs related to a PD occur in patients suffering from depression [29]. Concerning the cases with repeated instances of SRB, a similar pattern could be identified (OR = 6.2; 95% CI 3.8–10.4). Among those, depression was also frequent (OR = 1.4; 95% CI 1.0–1.9).

This aspect together with the increased usage of antidepressants and neuroleptics might explain the relatively high percentage of cases with a history instances of SRB exceeding the findings in literature [13, 28]. A PD and the usage of antidepressants and neuroleptics seem associated with repeated suicide attempts [21].

The strong association of substance use disorders and (recurrent) instances of SRB demonstrated in the literature [30–32] is also supported by the present study in which approximately one-third of the cases with repeated suicide-attempts had a suspected substance use disorder (OR = 2.5; 95% CI 1.6–4.0).

Furthermore, in line with existing data, the proportion of ethanol addiction is particularly high for cases with recurrent instances of SRB (OR = 2.8; 95% CI 1.6–5.0) [24].

Alongside the finding that the presence of a PD is associated with an increased risk of further SRB, the presence of an (ethanol) addiction seems to constitute a second risk factor for recurrent SRB. Additionally, this study showed that the use of neuroleptics and antidepressants is frequent for cases with repeated instances of SRB. When viewed together with the results of other studies—in particular the finding by Pfeifer et al., who demonstrated that overdose of tricyclic antidepressants carries a high mortality rate [5]—the results could serve to potentially identify high-risk patients at an early stage and to initiate appropriate preventive measures such as closer monitoring according to their needs. This could involve various strategies such as psychological intervention [11], assistance to maintain abstinence to reduce impulsivity, provision of improved follow-up [30, 33, 34] and safe prescribing practices. Our study identified women aged < 18 years and patients aged 18–44 years as particularly affected by suicidal behavior. Family doctors and pediatricians, who are usually the first point of contact for these patients as well as (adolescent) psychiatrists should be sensitized to a depressive mood or suicidal thoughts. Therefore, providing the practicing doctors with a comprehensive information on predictive factors for suicide could be an important preventive measure. Furthermore, since over-the-counter peripheral analgesics were the most often used substance group in patients aged 44 years or less, general prescription regulations for these substances and maximum package size should be reconsidered. These recommendations could also be extended to antidepressants and neuroleptics, which were the most frequently used substances in our study. Given that patient's own long-term medication was the predominant source of substances, both general practitioners and psychiatrists should reconsider their prescribing behavior, especially regarding the package size and repetitive prescriptions without personal patient contact.

Nevertheless, further research is needed, especially regarding psychosocial factors and demographic data, which can also have a decisive impact on SRB. These studies should also include other types of suicide, as well as differentiate between different degrees of severity of intoxication to detect particularly serious risk constellations. An accurate diagnosis and classification of PDs by a psychiatrist could also identify PDs with an exceptionally high SRB risk. Additionally, future studies should distinct between attempted suicide and completed suicide, to be able to better classify the data obtained on age, gender, substance use disorders and substances. Finally, our findings warrant confirmation by other studies which ultimately could pave the way towards development and adoption of better suicide prevention strategies.

## Study limitations

Although the cohort of this study is relatively large, this study has several important limitations. First, the study examined data from incoming calls in the real-life situation of acute PCC advisory. This means that information might be incomplete and no follow-up on the outcome of the individual showing SRB is available. For example, we did not distinguish between completed suicide and non-lethal suicide attempts. However, distinction between these two groups of patients is hardly possible due to a difficult follow-up of patients whose first contact was made through our PCC but a follow-up was not feasible. Second, given the setting of the study, we included only patients with self-poisoning and for this reason our findings cannot be directly extrapolated to patients with other SRB. Nevertheless, the information obtained within this study could be used to develop preventive measures against deliberate self-poisoning involving change to the prescription behavior and raising awareness about suicide among the physicians. Third, classification of psychiatric disorders was not always made by a specialist in psychiatry but on the basis of anamnestic information provided by the patient or his relatives, or by taking into account the existing medical records. In particular, substance use disorder was assigned by a board-certified psychiatrist only in 13 patients while in 161 patients, it was attributed based on the anamnestic information. Thus, substance use disorder was not exactly confirmed by the ICD criteria in the latter group, which could have resulted in an overestimation of the incidence of this psychiatric disorder. Given this limitation, we did not attempt to investigate which mental disorders could be associated with a high risk of SRB. Nevertheless, it should be noted that our study was performed in an unselected population; that is we included all incoming calls, irrespective of whether they concerned a patient with PD. Thus, our study also includes patients who did not receive (inpatient) psychiatric care due to a clinically asymptomatic or mild course as well as credibly assured distance from suicidality, because they were discharged immediately from the emergency department or after a short monitoring period. Since such patients are rarely included in studies on suicide conducted by psychiatrists, the findings of our study potentially reflect the SRB related to self-poisoning in a broad population. Moreover, for some variables, such as the reason for displaying SRB, data are available for only approximately half of the cases. Furthermore, we did not collect data on psychosocial factors or demographic characteristics, such as marital status and financial or employment status. Similarly, we did not collect the data on the comorbidities associated with the psychiatric disorders which precluded the association of these factors with SRB. Interpretation of the data has therefore to be carried out cautiously. Finally, our decision to use the nomenclature of Silverman et al. [4] may result in an overestimation of suicidal intoxications. This is due to the fact that in addition to patients with a suicidal intent, our study also included those with primarily self-injurious behavior, for example in the context of borderline personality disorder and patients who merely desire rest, sleep or freedom from pain by overdosing on medication. Nevertheless, these different intents are less relevant for the emergency and intensive care physician, at least for the initial care of the patient. In case of doubt, the treating physician usually assumes a certain intention to die, and this presumption is only verified later at the time of first psychiatric exploration.

## Conclusion

The study delineates several characteristics of individuals displaying SRB by means of self-intoxication based on data collected from an unselected population in the real-world scenario of a PCC. It identifies differences based on gender, age, and recurrence of instances of SRB factors that are not only of considerable importance for the development of preventive measures but also allow for individual adaptation and use of the measures. The analysis shows that different characteristics are associated with an increased risk of SRB particularly the risk for recurrent instances of SRB. Hence, our findings may serve as a basis for future research, both on a national and international level to verify the characteristics identified here and offer further guidance for preventive strategies of suicides.

## Supplementary Information

**Additional file 1:** Geith et al. 2021_Supplement 1. Lessons to be learned: Identifying high-risk medication and circumstances in patients at risk for suicidal self-poisoning—Modified poison emergency call record.

**Additional file 2:** Geith et al. 2021_Supplement 2. Lessons to be learned: Identifying high-risk medication and circumstances in patients at risk for suicidal self-poisoning—Pairwise comparison of reasons for SRB in different age groups.

**Additional file 3:** Geith et al. 2021_Supplement 3. Lessons to be learned: Identifying high-risk medication and circumstances in patients at risk for suicidal self-poisoning—Overview about literature research with respect to self-intoxication in correlation to age, gender, and substances used.

## Data Availability

All data generated or analyzed during this study are included in this published article. The datasets used and/or analyzed during the current study are available from the corresponding author on reasonable request.

## Abbreviations

ICD-10: International statistical classification of diseases and related health problems: 10th revision
IRB: Institutional review board
PCC: Poison Control Centre
PD: Psychiatric disorder
SRB: Self-poisoning on the ground of Suicide-Related Behavior
