# Supplementary material for: Lessons to be learned: identifying high-risk medication and circumstances in patients at risk for suicidal self-poisoning
Source: Int J Ment Health Syst. 2022 Jan 25;16:4. doi: 10.1186/s13033-021-00513-8 (PMC8788074; doi:10.1186/s13033-021-00513-8)
Supplement: Supplementary file 1 — Additional file 1: Geith et al. 2021_Supplement 1. Lessons to be learned: Identifying high-risk medication and circumstances in patients at risk for suicidal self-poisoning—Modified poison emergency call record. [file 13033_2021_513_MOESM1_ESM.pdf]

Lessons to be learned: Identifying high-risk medication and circumstances in patients at risk for suicidal self-poisoning

Geith, Stefanie, Didden, Christiane, Rabe, Christian, Zellner, Tobias, Ott, Armin, Eyer, Florian

Form sheet

Poison emergency call

Klinikum rechts der Isar   Technische Universität München

Code 2018

Answered by   forwarded to

___(Min.)

Caller: Layperson | Hospital | Surgery | Emergency | Pharmacy | Other | Inst

Amount (unk./minor)

Digital recording at pharmaceutical company **no** ☐

Poison / Manufacturer

Postal Code:

Co-ingestion with drug: Alcohol: unk. / no / yes
Illicit drug: unk./ no / yes, specify

Phone:

Patient name:
m / f

Age / **Birth date**:

Weight:

Enquiry: ☐

Ingestion:

Time:

Aetiology: home-rel. accident ☐ para-/ suicidal ☐ ☐ abuse ☐ commercial ☐ external force ☐ medical accident ☐ side effect ☐

Source: own medication ☐ medication from family / friends ☐ unknown ☐

medicine chest: ☐ own pharmacy ☐ several pharmacies ☐ mail order pharmacy ☐ unknown ☐

Case History:

Symptoms: ☐ none ☐ unknown ☐ yes:

Latency until appearance of symptoms:

Co-morbidities / Psychiatric Disease
unk./ no / yes, specify

Addiction disorder unk./no / yes, specify substance

Prior suicide attempt(s) unk/ no / yes
Emotional act (e.g.. dispute) unk./ no / yes
Childbirth within the last year unk./ no / yes

Comment:

☐ Physician ☐ Hospital ☐ at home

Consultation: ☐ gastric lavage ☐ vomition ☐ activated charcoal ☐ antidote ☐ defoaming agent ☐ liquid

Consent for further investigation for studies ☐ **no** ☐ **yes**, availability how (phone) who (if applicable, different contact person) when date / time

REMOVAL OF TOXIC SUBSTANCES
ANTIDOTE

FIRST AID

TRANSPORT

ASSERVATION

Invoice: yes/no how often?   Follow-Up: ☐   Consulting physician

Supplement 2: Modified poison emergency call record
