# Supplementary material for: Lessons to be learned: identifying high-risk medication and circumstances in patients at risk for suicidal self-poisoning
Source: Int J Ment Health Syst. 2022 Jan 25;16:4. doi: 10.1186/s13033-021-00513-8 (PMC8788074; doi:10.1186/s13033-021-00513-8)
Supplement: Supplementary file 2 — Additional file 2: Geith et al. 2021_Supplement 2. Lessons to be learned: Identifying high-risk medication and circumstances in patients at risk for suicidal self-poisoning—Pairwise comparison of reasons for SRB in different age groups. [file 13033_2021_513_MOESM2_ESM.pdf]

Lessons to be learned: Identifying high-risk medication and circumstances in patients at risk for suicidal self-poisoning

Geith, Stefanie, Didden, Christiane, Rabe, Christian, Zellner, Tobias, Ott, Armin, Eyer, Florian

	**< 18**	**18-44**	**45-64**
**18-44**	5.3e-05	-	0.00086
**45-64**	3.0e-09	0.00086	-
**>64**	0.00251	0.09791	0.02306

Supplement 2a: Pairwise comparison addictive substance and age groups.

	**< 18**	**18-44**	**45-64**
**18-44**	1.3e-05	-	-
**45-64**	8.8e-08	0.0421	-
**>64**	0.0315	0.0610	0.0029

Supplement 2b: Pairwise comparison addiction and age groups.

	**< 18**	**18-44**	**45-64**
**18-44**	3.7e-05	-	-
**45-64**	0.00012	0.96986	-
**>64**	5.9e-05	0.41108	0.41041

Supplement 2c: Pairwise comparison gender and age groups

	**< 18**	**18-44**	**45-64**
**18-44**	0.8095	-	-
**45-64**	0.6581	0.7926	-
**>64**	0.0088	4.1e-05	8.2e-05

Supplement 2d: Pairwise comparison PD and age groups.

	**< 18**	**18-44**	**45-64**
**18-44**	0.0809	-	-
**45-64**	0.5351	0.2097	-
**>64**	0.3792	0.0042	0.0822

Supplement 2e: Pairwise comparison recurrent suicide attempts and age groups.

	**< 18**	**18-44**	**45-64**
**18-44**	0.2672	**-**	**-**
**45-64**	0.0064	0.0144	**-**
**>64**	0.0837	0.2703	0.7448

Supplement 2f: Pairwise comparison peripheral analgesics and age groups.

	**< 18**	**18-44**	**45-64**
**18-44**	1.0000	**-**	**-**
**45-64**	0.7113	0.4170	**-**
**>64**	0.0401	0.0087	0.0486

Supplement 2g: Pairwise comparison neuroleptics and age groups.

	**< 18**	**18-44**	**45-64**
**18-44**	0.704	**-**	**-**
**45-64**	0.200	0.126	**-**
**>64**	0.033	0.018	0.289

Supplement 2h: Pairwise comparison anesthetic analgesics and age groups.

	**< 18**	**18-44**	**45-64**
**18-44**	0.0051	**-**	**-**
**45-64**	0.0016	0.4290	**-**
**>64**	4.6e-05	0.0152	0.1114

Supplement 2i: Pairwise comparison benzodiazepine and age groups.

	< 18	18-44	45-64
**18-44**	0.0232	-	-
**45-64**	0.0021	0.0694	-
**>64**	0.0019	0.1107	0.8616

Supplement 2j: Pairwise comparison Z-drugs and age groups.

	< 18	18-44	45-64
**18-44**	0.0002	-	-
**45-64**	6.5e-06	0.0769	-
**>64**	5.9e-08	0.0010	0.0807

Supplement 2k: Pairwise comparison Benzodiazepines / Z-drugs and age groups.

	< 18	18-44	45-64
**18-44**	0.088	-	-
**45-64**	0.466	5.8e-05	-
**>64**	0.216	7.7e-05	0.489

Supplement 2l: Pairwise comparison cardiovascular drugs and age groups.

	< 18	18-44	45-64
**18-44**	0.00012	-	-
**45-64**	7.9e-07	0.06857	-
**>64**	1.2e-05	0.04003	0.44713

Supplement 2m: Pairwise comparison own medication as source and age groups.

	< 18	18-44	45-64
**18-44**	0.137	-	-
**45-64**	0.002	0.017	-
**>64**	0.617	0.543	0.026

Supplement 2n: Pairwise comparison medicine chest as source and age groups.

	< 18	18-44	45-64
**18-44**	0.00019	-	-
**45-64**	0.00026	0.76377	-
**>64**	0.00138	0.76377	0.76377

Supplement 2o: Pairwise comparison family and friends as source and age groups.

	< 18	18-44	45-64
**18-44**	1.0e-11	-	-
**45-64**	4.5e-12	0.33	-
**>64**	0.03	1.7e-05	6.2e-06

Supplement 2p: Pairwise comparison co-ingestion and age groups.

	< 18	18-44	45-64
**18-44**	0.119	-	-
**45-64**	0.025	0.667	-
**>64**	7.5e-07	3.9e-06	1.7e-05

Supplement 2q: Pairwise comparison reason for suicide attempt and age groups.

Supplement 2: Age group: Pairwise comparisons of proportions. Adjustments for p-value: none.
