# Supplementary material for: Lessons to be learned: identifying high-risk medication and circumstances in patients at risk for suicidal self-poisoning
Source: Int J Ment Health Syst. 2022 Jan 25;16:4. doi: 10.1186/s13033-021-00513-8 (PMC8788074; doi:10.1186/s13033-021-00513-8)
Supplement: Supplementary file 3 — Additional file 3: Geith et al. 2021_Supplement 3. Lessons to be learned: Identifying high-risk medication and circumstances in patients at risk for suicidal self-poisoning—Overview about literature research with respect to self-intoxication in correlation to age, gender, and substances used. [file 13033_2021_513_MOESM3_ESM.pdf]

Lessons to be learned: Identifying high-risk medication and circumstances in patients at risk for suicidal self-poisoning

Geith, Stefanie, Didden, Christiane, Rabe, Christian, Zellner, Tobias, Ott, Armin, Eyer, Florian

Author/Year	Study design	Country	Sample size	Median age in years (range)	Male	Female	Main substances used
Hendrix L et al., 2012 (1)	Retrospective study	Belgium	312	37	122 (39.1%)	190 (60.9%)	Benzodiazepine (51%), antidepressants (23%), antipsychotics (16%)
Prescott K et al., 2009 (2)	Retrospective study	UK	2665	32 (16-90)	40.8%	59.2%	Paracetamol (43%), ibuprofen (17%).
Bilén K et al., 2011 (3)	Retrospective study	Sweden	1524	39.5	35%	65%	Benzodiazepine (45%), antidepressants (16%), paracetamol (16%)
Kordrostami et al., 2017 (4)	Forensic toxicology analysis	Iran	674 (completed suicides)	32.61	462 (68.55%)	212 (31.45%)	Aluminium phosphide tablets (pesticides), opioids, methamphetamine
Salles et al., 2018 (5)	Retrospective study	France	516	42	165 (31.9%)	351 (68.1%)	Benzodiazepine (85%), paracetamol (9%), opioids (4%)
Marahatta et al., 2009 (6)	Prospective study	Nepal	54	29.87 (females), 35.54 (males)	42.6%	57,4%	Organophosphorus (74%), antidepressants (17%)
Mauri et al., 2005 (7)	Prospective study	Italy	201	40	73 (36%)	128 (64%)	Benzodiazepine (59%), neuroleptics (13%), antidepressants (13%)
Cook et al., 2008 (8)	Prospective study	UK, Scotland	530	33	35%	65%	Paracetamol (39%), antidepressants (35%), hypnotics/anxiolytics (25%)
Ghazinour et al., 2009 (9)	Prospective study	Iran	2025 (parasuicides)	25.4 (females), 28.5 (males)	966 (47.7%)	1059 (52.3%)	Psychotropic drugs and anticonvulsants (56%)
Sorge et al., 2015 (10)	Prospective study	Germany	3533	35 (males), 29 (females)	62.6%	37.4%	Benzodiazepine (29%), antidepressants (20%), antihistamines (13%)
Michel et al., 1994 (11)	Prospective study	Switzerland	Completed suicide: 179 Attempted suicide: 269	Completed suicide: 53 Attempted suicide: 36	Completed suicide: 49% Attempted suicide: 35%	Completed suicide: 51% Attempted suicide: 65%	Benzodiazepine (CS: 44%, AS: 46%), antidepressants (CS: 16%, AS: 11%)

Supplement 3: Overview about literature research with respect to self-intoxication in correlation to age, gender, and substances used

References

1. Hendrix L, Verelst S, Desruelles D, Gillet JB. Deliberate self-poisoning: characteristics of patients and impact on the emergency department of a large university hospital. Emerg Med J [Internet]. 2013 Jan [cited 2021 Nov 22];30(1). Available from: https://pubmed.ncbi.nlm.nih.gov/22328636/

2. Prescott K, Stratton R, Freyer A, Hall I, Le Jeune I. Detailed analyses of self-poisoning episodes presenting to a large regional teaching hospital in the UK. Br J Clin Pharmacol [Internet]. 2009 [cited 2021 Sep 23];68(2):260–8. Available from: https://pubmed.ncbi.nlm.nih.gov/19694747/

3. Bilén K, Ottosson C, Castrén M, Ponzer S, Ursing C, Ranta P, et al. Deliberate self-harm patients in the emergency department: factors associated with repeated self-harm among 1524 patients. Emerg Med J [Internet]. 2011 Dec [cited 2021 Nov 22];28(12):1019–25. Available from: https://pubmed.ncbi.nlm.nih.gov/21076053/

4. Kordrostami R, Akhgari M, Ameri M, Ghadipasha M, Aghakhani K. Forensic toxicology analysis of self-poisoning suicidal deaths in Tehran, Iran; trends between 2011-2015. DARU J Pharm Sci [Internet]. 2017 Jun 13 [cited 2021 Nov 27];25(1). Available from: /pmc/articles/PMC5470324/

5. Salles J, Calonge J, Franchitto N, Bougon E, Schmitt L. Factors associated with hospitalization after self-poisoning in France: Special focus on the impact of alcohol use disorder. BMC Psychiatry [Internet]. 2018 Sep 6 [cited 2021 Nov 22];18(1):1–9. Available from: https://bmcpsychiatry.biomedcentral.com/articles/10.1186/s12888-018-1854-0

6. Marahatta SB, Singh J, Shrestha R, Koju R. Poisoning cases attending emergency department in Dhulikhel Hospital- Kathmandu University Teaching Hospital. Kathmandu Univ Med J (KUMJ) [Internet]. 2009 [cited 2021 Nov 27];7(26):152–6. Available from: https://pubmed.ncbi.nlm.nih.gov/20071851/

7. Mauri MC, Cerveri G, Volonteri LS, Fiorentini A, Colasanti A, Manfré S, et al. Parasuicide and drug self-poisoning: analysis of the epidemiological and clinical variables of the patients admitted to the Poisoning Treatment Centre (CAV), Niguarda General Hospital, Milan. Clin Pract Epidemiol Ment Health [Internet]. 2005 Apr 28 [cited 2021 Nov 22];1(1). Available from: https://pubmed.ncbi.nlm.nih.gov/15967050/

8. Cook R, Allcock R, Johnston M. Self-poisoning: current trends and practice in a U.K. teaching hospital. Clin Med [Internet]. 2008 [cited 2021 Nov 22];8(1):37–40. Available from: https://pubmed.ncbi.nlm.nih.gov/18335667/

9. Ghazinour M, Emami H, Richter J, Abdollahi M, Pazhumand A. Age and gender differences in the use of various poisoning methods for deliberate parasuicide cases admitted to loghman hospital in Tehran (2000-2004). Suicide Life Threat Behav [Internet]. 2009 Apr [cited 2021 Nov 22];39(2):231–9. Available from: https://pubmed.ncbi.nlm.nih.gov/19527164/

10. Sorge M, Weidhase L, Bernhard M, Gries A, Petros S. Self-poisoning in the acute care medicine 2005-2012. Anaesthesist [Internet]. 2015 Jun 22 [cited 2021 Nov 22];64(6):456–62. Available from: https://pubmed.ncbi.nlm.nih.gov/25951922/

11. Michel K, Arestegui G, Spuhler T. Suicide with psychotropic drugs in Switzerland. Pharmacopsychiatry [Internet]. 1994 [cited 2021 Nov 27];27(3):114–8. Available from: https://pubmed.ncbi.nlm.nih.gov/8078951/
